# Conservation of parasites: A primer

**DOI:** 10.1016/j.ijppaw.2023.07.001

**Published:** 2023-07-03

**Authors:** Alan J. Lymbery, Nico J. Smit

**Affiliations:** a Centre for Sustainable Aquatic Ecosystems, Harry Butler Institute, Murdoch University, Murdoch, 6150, Western Australia, Australia; b Water Research Group, Unit for Environmental Sciences and Management, North-West University, Private Bag X6001, Potchefstroom, 2520, South Africa

**Keywords:** Biodiversity, Intrinsic value, Ecosystem service, Bioindicator, Threatened species, Conservation management

## Abstract

Although parasites make up a substantial proportion of the biotic component of ecosystems, in terms of both biomass and number of species, they are rarely considered in conservation planning, except where they are thought to pose a threat to the conservation of their hosts. In this review, we address a number of unresolved questions concerning parasite conservation. Arguments for conserving parasite species refer to the intrinsic value conferred by their evolutionary heritage and potential, their functional role in the provision of ecosystem services, and their value as indicators of ecosystem quality. We propose that proper consideration of these arguments mean that it is not logically defensible to automatically exclude parasite species from conservation decisions; rather, endangered hosts and parasites should be considered together as a threatened ecological community. The extent to which parasites are threatened with extinction is difficult to estimate with any degree of confidence, because so many parasite species have yet to be identified and, even for those which have been formally described, we have limited information on the factors affecting their distribution and abundance. This lack of ecological information may partially explain the under-representation of parasites on threatened species lists. Effective conservation of parasites requires maintaining access to suitable hosts and the ecological conditions that permit successful transmission between hosts. When implementing recovery plans for threatened host species, this may be best achieved by attempting to restore the ecological conditions that maintain the host and its parasite fauna in dynamic equilibrium. Ecosystem-centred conservation may be a more effective strategy than species-centred (or host-parasite community-centred) approaches for preventing extinction of parasites, but the criteria which are typically used to identify protected areas do not provide information on the ecological conditions required for effective transmission. We propose a simple decision tree to aid the identification of appropriate conservation actions for threatened parasites.

## Highlights

• Parasites are common in all ecosystems.
• Parasites should be conserved for their intrinsic value and ecosystem services.
• Parasites are usually regarded only as threats by conservation managers.
• A decision tree for integrating parasites into conservation planning is proposed.

## Graphical abstract

**Figure:**
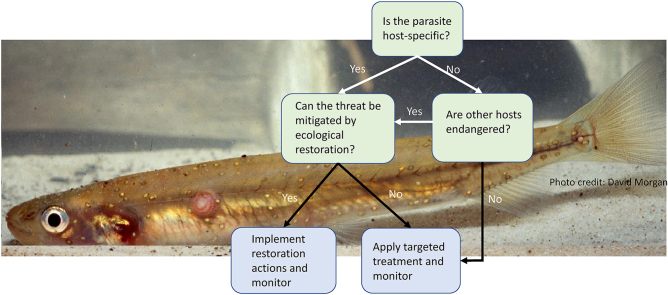

## 1 Introduction

The Californian condor (*Gymnogyps californianus*) conservation project is often hailed as an iconic conservation success story. Although once wide-ranging throughout North America, by 1982 less than 30 birds remained in the wild. These birds formed the basis of a captive breeding and release program that has seen the numbers increase to over 300 wild birds and approximately 200 in captivity (Robinson et al., 2021). This has been achieved with a substantial financial commitment; the total cost of the project to 2008 was estimated to be USD 45 million (https://www.science.org/content/article/condor-rescue-program-danger-failure), with ongoing annual costs of USD 5 million (Walters et al., 2010). It has also come at the cost of extinction of a host-specific louse (*Colpocephalum californici*), believed to be deliberately killed during the establishment of the condor captive breeding colony (Dunn, 2009). The net return on an investment of approximately USD 115 million over 35 years is therefore one free-living species saved and one parasitic species lost. Is this how conservation should work?

Parasites are common in all ecosystems, in terms of both number of species (Dobson et al., 2008) and biomass (Kuris et al., 2008). There is now also a substantial body of research testifying to the importance of parasites in maintaining ecosystem function, through direct and indirect effects on free-living organisms (Preston et al., 2016). Despite their ubiquity and importance, however, parasites are poorly represented in listings of threatened species and usually ignored in conservation planning, except, as in the case of the California condor, as threats to the conservation of their hosts.

Calls for the conservation of parasite species began almost 30 years ago (Windsor, 1995; Durden and Keirans, 1996) and have continued, albeit sporadically, ever since (e.g. Gompper and Williams, 1998; Pizzi, 2009; Moir et al., 2012; Gómez and Nichols, 2013; Dougherty et al., 2016; Carlson et al., 2020a). While many of these studies provide general guidelines to advance the conservation of parasites, little progress has been made in implementing practical conservation plans for endangered parasite species. This can be partly explained by the under-representation of parasites in conservation textbooks and courses, and the largely negative perception of them by conservation practitioners (Nichols and Gómez, 2011; Carlson et al., 2020a). It is also due, however, to inconsistencies in our understanding of parasite biodiversity and how to conserve it, such as the rationale for parasite conservation, the number of parasite species that are likely to be threatened with extinction, and which of these should be prioritized for conservation. In this review, we focus on these knowledge gaps, which we believe need to be considered more fully if we are to move from general concern about parasite conservation to the integration of parasite species in conservation policy and practice.

## 2 Why should we conserve parasites?

Arguments for conserving parasite species fall into three (not mutually exclusive) categories: 1) parasites should be conserved because they are part of natural ecosystems, no less than the charismatic vertebrates to which most conservation research and funding is directed; 2) parasites should be conserved because they play vital roles in maintaining ecosystem organization and function; 3) parasites should be conserved because they are useful bioindicators of ecosystem health. These arguments mirror an ongoing tension in the conservation literature between emphasizing the intrinsic (moral or existence) value of species and ecosystems, i.e. as ends themselves; or concentrating on their utilitarian (instrumental) value, through the provision of goods and services that benefit humans (Loreau, 2014; Batavia and Nelson, 2017).

There are many philosophical complexities to the concept of intrinsic value (Batavia and Nelson, 2017), but the primary distinction for conservation biologists is between subjective and objective intrinsic value. Subjective intrinsic value is created by people and is contingent upon their attitudes or beliefs (Callicot, 1986). Consequently, some species can have more subjective intrinsic value than others, and different people will rank their relative values differently. Objective intrinsic value, by contrast, is not conferred by people, but exists in species (and other natural entities) because of their evolutionary heritage and potential (Rolston, 1988). The objective intrinsic value of a species is therefore independent of any relationship it may have with humans and cannot be ranked against the intrinsic value of other species. Objective intrinsic value was integral to the development of the modern science of conservation biology, from the land ethic of Aldo Leopold (1949) to the articulation of the field by Michael Soulé (1985), and is enshrined as the primary belief value of the Society for Conservation Biology: “There is intrinsic value in the natural diversity of organisms, the complexity of ecological systems, and the resilience created by evolutionary processes” (https://conbio.org/about-scb/who-we-are/). From this perspective, parasites are just as deserving of protection as their hosts, so long as they are equally endangered (accepting, of course, that hosts must be protected if parasites are not to become extinct). This is the point of view espoused by, among others, Windsor (1995) and Gompper and Williams (1998).

Although many conservation researchers and managers still profess a commitment to intrinsic value (Berry et al., 2018), in the last two decades its importance in conservation practice has been largely supplanted by an approach which emphasises the utilitarian value of species, communities and ecosystems, particularly through the provision of ecosystem services (Batavia and Nelson, 2017). Ecosystem services are those ecological functions that benefit human life and well-being, where ecological function refers to the flow of energy and materials through the biotic and abiotic components of an ecosystem. Ecosystem services are typically categorized as supporting (those necessary for the production of all other services, e.g. oxygen production, soil formation, nutrient cycling), provisioning (products obtained directly from ecosystems, such as food and fresh water), regulating (those that maintain ecosystem structure or regulate ecosystem processes, such as climate regulation and food web stability) or cultural (non-material benefits, such as recreation and spiritual or aesthetic enrichment) (Alcamo et al., 2003).

The ecosystem services approach arose from concern that an emphasis on intrinsic value lacked practical power in generating public and government support for conservation (Tallis and Lubchenco, 2014). This has particular resonance for parasite conservation, given the negative perception that most people have of parasites (Gómez and Nichols, 2013). Understandably, therefore, most arguments for parasite conservation have emphasised their functional role in maintaining the health of individual hosts, host populations and entire ecosystems (e.g. Nichols and Gómez, 2011; Dougherty et al., 2016; Selbach et al., 2022). For individual hosts, parasites impose energetic demands that reduce growth, fecundity and survival (Marcogliese, 2004), but these adverse impacts on host fitness may be offset by positive effects, for example promoting the proper functioning of the host immune system (Stringer and Linklater, 2014; Spencer and Zuk, 2016) and providing protection against pollutants such as heavy metals (Sures, 2003; Sánchez et al., 2016). Even when their net effect is to decrease the fitness of individual hosts, parasites may play important roles in maintaining the long-term viability of host populations by regulating host population size (Hudson et al., 1998) and increasing genetic diversity (Coltman et al., 1999). At higher levels of organisation, parasites may mediate predatory and competitive interactions among free-living species, shaping community structure and diversity, food web complexity and energy flow through the ecosystem (Hudson et al., 2006; Dunne et al., 2013; Hatcher et al., 2014; Vannatta and Minchella, 2018). Through these processes, parasites play important, although often unpredictable roles in ecosystem functioning and therefore contribute in diverse ways to the provision of ecosystem services, particularly supporting and regulating services (Preston et al., 2016; Frainer et al., 2018).

Bioindicators (i.e. biological processes, species or communities that are used to assess changes in environmental quality) have gained increasing importance in conservation and natural resource management (Burger, 2006). The value of parasites as bioindicators of environmental health is now well established within the sub-discipline of environmental parasitology that focuses on the interactions that exist between parasites and environmental pollutants (Marcogliese, 2005; Sures et al., 2017). The usefulness of parasites in monitoring anthropogenic impacts stems from the ability of certain groups, such as acanthocephalans, cestodes and nematodes, to accumulate pollutants at much higher levels than their host (Erasmus et al., 2020; Nachev et al., 2022). Furthermore, the complex life cycles of many heteroxenous parasites, such as trematodes, make them sensitive to environmental change, influencing parasite abundance and diversity directly through mortality, or indirectly through the loss of suitable intermediate hosts (Sures and Nachev, 2022). In a recent review on the effects of stressors on aquatic parasites, Sures et al. (2023) showed that habitat alteration, global warming, and pollution are some of the main stressors that impact parasites. Parasites, owing to their intricate interplay with these stressors, serve as a reflection of the state and complexity of ecosystems and therefore have the potential to function as bioindicators, facilitating the evaluation of environmental conditions (Sures et al., 2023).

## 3 How many parasites are threatened with extinction?

Since determining the conservation status of parasite species is still an emerging field of research, there are very limited data available on the extent to which parasites are endangered (see Section 5.1). A major limiting factor to obtaining reliable estimates of parasite threat status is our incomplete knowledge of parasite diversity; parasites are thought to account for between 30% and 50% or more of all living species, but the relatively less advanced state of parasite taxonomy (compared to their hosts) and the dramatic recent increase in cryptic species uncovered by molecular techniques means that we are very far from a complete inventory of parasite biodiversity (Poulin, 2014). Most studies which have attempted to describe parasite diversity have extrapolated from estimates of the number of parasite species per host species and their host specificity for the better-known groups of parasites from vertebrate hosts. For example, taking previous work from Poulin and Morand (2004) into consideration, Dobson et al. (2008) estimated that there are between 75,000 and 300,000 species of parasitic helminths (trematodes, cestodes, nematodes and acanthocephalans) of vertebrates. Twelve years later Carlson et al. (2020b) estimated between 100,000 and 350,000 species, with 85–95% still unknown, underscoring the potential to lose through extinction many parasite species that have not yet been identified or classified.

The most obvious cause of parasite extinction, at least for parasites with high host-specificity, is coextinction; the extinction of a parasite species as a consequence of its dependence on a host that has become extinct (Dunn et al., 2009). For host-specific parasites, conservation status of the host has therefore been used as an indication of endangerment. Koh et al. (2004) predicted potential future extinctions (if all threatened host species went extinct) of 593 monogenean parasites of fishes, 342 lice and 193 mites of birds, and 20 nematodes and 12 lice of primates. Using similar methodology, Mihalca et al. (2011) proposed one co-extinct and 63 co-endangered hard ticks of reptiles, birds and mammals; Rózsa and Vas (2014) listed six co-extinct and 40 critically co-endangered lice of birds and mammals, with another two to four species that went extinct as a result of conservation efforts to save the host; and Kwak (2018) assigned one flea species parasitising Australian mammals as co-extinct and seven as threatened with extinction. On a broader scale, Dobson et al. (2008) suggested that between 3% and 5% of all the estimated species of parasitic helminths of vertebrates will be threatened with extinction in the next 50–100 years. In a recent study on the parasites of threatened freshwater mussels of the families Unionidae and Margaritiferidae, Brian and Aldridge (2023) estimated that 60 parasite species (21% of the total parasite fauna) of these mussels are at immediate risk of extinction, clearly showing the risk to parasites of threatened host species.

These estimates of parasite extinction risk, sobering as they are, almost certainly underestimate the true extent of endangerment. Host-specific parasites are likely to be more endangered than their hosts, because a decline in host population size prior to extinction may reduce transmission levels below the threshold required for maintenance of the parasite population, particularly as parasites are typically over-dispersed among host individuals (Shaw and Dobson, 1995). In addition, parasites will be susceptible to many of the same threats affecting free-living species, such as habitat loss, invasive species and climate change, either by acting directly on free-living stages or by altering the physiology, behaviour or ecology of their hosts (Colwell et al., 2012). These threats may pose a particular risk to host generalists and parasites with complex life cycles if they decrease host geographic range and cross-species contact rates (Lafferty, 2012; Farrell et al., 2015). In an extensive study using 53,133 occurrence records of the geographic ranges of 457 parasite species, Carlson et al. (2017a) predicted that due to climate-driven habitat loss, 5–10% of these species will potentially be extinct by 2070. They also calculated that coextinction alone would result in the loss of 8–24% of parasite species.

A recently proposed area of research, known as the Historical Ecology of Parasitism (HEP), aims to determine whether parasite populations are increasing or decreasing using natural history collections (Wood and Vanhove, 2023; Wood et al., 2023a). Although using natural history collections to gather information on parasite ecology is not novel (Welicky et al., 2019), the advantage of employing the HEP approach is its potential to generate data on parasite biodiversity loss (Wood et al., 2023b). Such data can be an essential tool in parasite conservation efforts, as it can help identify declining parasite populations and the potential drivers of changes in parasite abundance.

## 4 Which parasites should be conserved?

A dilemma which confronts all proponents of parasite conservation is the appropriate balance between conserving parasites and protecting the health and well-being of their hosts. Parasites which infect people and livestock have major effects on human mortality, morbidity and economic security (Kuris, 2012; Rist et al., 2015). Parasitic disease has also been identified as an important cause of wildlife declines, particularly when host populations are affected by other factors such as habitat degradation or loss of genetic diversity, or when exotic parasites are introduced (Holmes, 1996; Dunn et al., 2012; Edworthy et al., 2019). It is often necessary, therefore, to exclude certain parasite species from considerations of conservation. The global parasite conservation plan of Carlson et al. (2020a), for example, explicitly excludes all microparasites and those macroparasites that (a) are a known or suspected risk to human health or livelihoods, or (b) threaten the conservation of their (wildlife) hosts, unless alternate hosts or ex situ preservation can be used. Although we agree with this general principle, in practice deciding which parasites should be excluded from considerations of conservation might not be so straightforward, for several reasons.

Firstly, although we possess a relatively good understanding of parasites that pose a risk to human health and there is an obvious need to prioritize the health of people over considerations of parasite conservation, our understanding of parasites that affect livelihoods, especially with regards to parasites of recently domesticated livestock species, is not as clear. Cucchi and Arbuckle (2021) indicate that domestication of animals began with dogs 23,000 years ago and was followed 10,000 years later by the domestication of globally important livestock such as sheep, goats, pigs, and cattle, resulting in a good knowledge base of the parasites affecting these animals. However, with the human population projected to reach 10 billion by 2050, the demand for animal protein is ever-increasing, leading to ongoing domestication of new species, especially fish, which is a rapidly growing field with almost 100 species currently considered as domesticated (Teletchea, 2021). The selection of new fish species for aquaculture, particularly native species, is driven by the need to create a more diverse and resilient aquaculture sector in the face of environmental change (Teletchea, 2021). However, this creates a major challenge for the conservation of aquatic parasites, as the selection of new fish species can potentially create conflict between parasite conservation efforts and the control of parasitic diseases in aquaculture. Fish hosts in their natural habitats harbour a range of parasites with varying degrees of specificity, but some parasites have a notable capacity to proliferate in aquaculture settings (Buchmann, 2022). As new fish species are constantly selected for domestication in both freshwater and marine finfish aquaculture, the selection of parasites for conservation becomes complicated, as there is uncertainty about whether the host may become a target species for aquaculture in the future, and whether its natural parasite species may cause disease when infection levels reach high intensities in the confined aquaculture environment.

The second problem we face in excluding, *a priori*, certain parasites from conservation actions is that parasite species are neither inherently harmful nor inherently benign. Pathogenicity (potential to cause disease) and virulence (degree of pathogenicity) are emergent properties of the interaction between parasite, host and environment (Casadevall et al., 2011; Garcia-Solache et al., 2013). This implies that the outcome of parasitic infection is often unpredictable, blurring the distinction between parasitism, commensalism and mutualism (Hirt, 2019) and in many cases making it difficult to accurately assess the likelihood and consequences of infection to human, livestock and wildlife health. A precautionary approach would exclude from conservation any parasites that present a disease risk, however small, to human health or livelihood, or to endangered wildlife hosts (Dougherty et al., 2016; Carlson et al., 2020a). However, while this might be ethically acceptable for parasites that threaten human health and livelihood, it could have consequences that are counterproductive for the conservation of endangered wildlife.

As already discussed, parasites may play vital regulatory roles for populations and communities of free-living animals, even when they reduce the fitness of individual hosts, so loss of parasite species, either through active eradication or lack of intervention to prevent extinction, may have unintended consequences. For example, using ivermectin to experimentally reduce nematode infections in white-footed mice (*Peromyscus leucopus*) resulted in a reciprocal increase in the prevalence of other gastrointestinal parasites, particularly coccidia, which have been associated with decreased body weight and reduced survival of the host (Pedersen and Antonovics, 2013). Similarly, anthelmintic treatment (pyrantel pamoate) of yellow-necked mice (*Apodemus flavicollis*) to reduce infections of the nematode *Heligmosomoides polygyrus* increased infestations of the tick *Ixodes ricinus*, which is a vector for tick-borne encephalitis virus (Ferrari et al., 2009). Such unintended consequences of parasite removal are not limited to the disruption of the parasite community within individual hosts but may also have much wider ecological impacts. When grey wolves (*Canis lupus*) were reintroduced to Yellowstone National Park, they were treated to remove a broad range of parasites, including the use of praziquantel against cestodes such as *Echinococcus granulosus* (Fritts et al., 1997). While *E. granulosus* is an important zoonosis in some areas of the world where it cycles between livestock and domestic dogs, it has little pathogenic effect on canid definitive hosts and by increasing predation risk for wildlife intermediate hosts, plays an important role in the ecology of threatened apex predators, such as wolves (Joly and Messier, 2004).

Finally, we believe that not considering conservation of a parasite species because it may pose an extinction risk to a wildlife host is not logically defensible on either intrinsic value or utilitarian grounds and in practice, will usually not be necessary. Objective intrinsic value is independent of human perception and is, therefore the same for parasites and hosts. From a utilitarian perspective, any choice between host and parasite conservation needs to be made on their relative value in providing ecosystem services. Considering the multiple, complex ways in which parasites may influence ecosystem functioning, it is quite possible, for example, that conserving a threatened, generalist parasite may contribute more to ecosystem services than conserving a rare, threatened host. Rare species are likely to contribute little to ecosystem services unless they perform unique functional roles or contribute disproportionately through indirect interactions, for example by mediating competition among service providers (Dee et al., 2019). A generalist parasite species which mediates competition, thereby increasing species evenness among free-living species in the ecosystem, is likely to have a much greater influence on service provision than one its rare hosts (Frainer et al., 2018). Luckily, in practice we are unlikely to be faced with such a stark choice between the conservation of a threatened host or a threatened parasite, because it should be possible to manage both as a threatened ecological community (see Section 5.2.2).

## 5 How will parasite conservation work in practice?

Conservation research and practice has a well-documented taxonomic bias in favour of taxa which are phylogenetically more closely related to humans or have anthropomorphic features (Clark and May 2002; Adamo et al., 2022). For example, an analysis of the flagship funding program for environment and climate action in the European Union (the LIFE program) found that investment in the conservation of the 1800 species of vertebrates in Europe was six times greater than for the 130,3000 species of invertebrates; in relative terms a 468 times greater investment in vertebrate species than invertebrate species (Mammola et al., 2020). This taxonomic bias is especially acute for parasite conservation, because parasites are almost always perceived negatively by both the general public (Barua et al., 2012) and conservation researchers and managers (Nichols and Gómez, 2011). As a consequence, despite the increasing interest in conservation among parasitologists and the development of general parasite conservation strategies (Dougherty et al., 2016; Carlson et al., 2020a), parasites are massively under-represented in threatened species lists, and there is a lack of specific conservation management plans (Stringer and Linklater, 2014; Kwak et al., 2019).

### 5.1 Threatened species lists

Threatened species lists provide an assessment of the probability of species extinction and can be produced at international or regional (national or local) scales. Despite the well-documented limitations of such lists for setting priorities for conservation actions and determining resource allocation (Possingham et al., 2002; Farrier et al., 2007), they remain a widely used policy and public relations tool for the conservation of biodiversity (Farrier et al., 2007; Moir and Brennan, 2020). The most comprehensive threatened species list is the Red List of the International Union for Conservation of Nature (IUCN). This assigns assessed species to one of eight different categories (Extinct, Extinct in the Wild, Critically Endangered, Endangered, Vulnerable, Near Threatened, Least Concern, Data Deficient) based on evaluation against five criteria: reduction in population size; declining, fragmented or fluctuating geographic range; small and declining population size; populations very small or restricted in distribution; and quantitative analysis of extinction risk (IUCN Standards and Petitions Committee, 2022). The Red List criteria provide a standardized, transparent approach to assessing conservation status and have been used to guide regional threatened species lists throughout the world (Miller et al., 2007; Brito et al., 2010).

Parasites are very rarely included in threatened species lists. Among the most important macroparasites of wildlife, for example, the IUCN Red List has no platyhelminths, nematodes or acanthocephalans and only one arthropod, the pygmy hog louse (*Haematopinus oliveri*; Critically Endangered). This may be largely a consequence of the requirement for data on species’ distribution and population trends to meet the IUCN criteria for listing, which are very often not available and difficult to obtain for invertebrates (Cardaso et al., 2011) and especially so for parasites and other dependent species which receive comparatively little research effort (Carlson et al., 2017b; Moir and Brennan, 2020; Poulin et al., 2023). However, the lack of parasites with Data Deficient status in the Red List, a category designed for taxa when there is inadequate information on which to make a sound assessment of distribution or population status, suggests that there is also insufficient recognition of parasites as integral components of biodiversity, rather than just as threats to the conservation of their hosts.

Modifications to the IUCN criteria for threatened species listing have been proposed, to enable the threat status of parasites and other dependent taxa to be defined by reference to the threat status of their host(s) (Mihalca et al., 2011; Kwak, 2018; Moir and Brennan, 2020; Barrera et al., 2022). This is supported by the IUCN guidelines in their definitions of population and population size (see pages 25 and 26; IUCN Standards and Petitions Committee, 2022), but is not specifically identified within the criteria themselves. In addition, reliance on the threat status of the host is likely to underestimate the true extinction risk of parasite species, which are typically over-dispersed within the host population, so Kwak et al. (2020) proposed a further modification, limiting the assessment of host range, population size and extinction risk to infected (or infested) hosts only, rather than all hosts. Application of these modified criteria requires data on prevalence, which is likely to be lacking and difficult to obtain, especially for endoparasites of threatened wildlife hosts, although increasing application of genomic sequencing of blood and faecal samples will help to overcome this limitation (e.g. Cooper et al., 2018; Zahedi et al., 2023), particularly as more parasite species are barcoded for identification. Carlson et al. (2020a) suggested the formation of an IUCN Working Group on Parasites and this has now been established (see https://iucn.org/our-union/commissions/group/iucn-ssc-parasite-specialist-group). This is a positive initiative which should help to formalize modifications to the criteria and streamline the listing process for parasite taxa. Other approaches to address the under-representation of parasites in threatened species lists include the formation of parasite-specific lists (Carlson et al., 2017b) and listing at the national or regional level, which has been achieved for the New England medicinal leech, *Macrobdella sesteria* in South Carolina and Massachusetts (Carlson and Phillips, 2020).

### 5.2 Conservation management

Effective conservation management requires a clear conservation target (e.g. a species, population, community or ecosystem) and a focus on the processes that support the dynamic requirements of that target (Pickett et al., 1992). If parasites are the conservation target, this means maintaining access to appropriate hosts and the ecological conditions that permit transmission from one host to the next. For a threatened parasite species this can be achieved, in theory, by a species-specific recovery plan and/or the protection of an entire ecosystem and the species it contains. Although there has long been debate over the relative merits of species-centred and ecosystem-centred approaches to conservation (Noss, 1996; Simberloff, 1998; Greene, 2005), in practice they are complementary, with each approach having strengths and weaknesses. Ecosystem-centred conservation may provide a more cost-effective way of protecting a large number of species with a limited budget, and deal more effectively with higher-order ecological processes and functions (Keith, 2009), but often lacks the detail needed to address factors causing the decline of individual species, especially those with complex life cycles or specialized requirements, such as parasites (Lindenmayer et al., 2007).

#### 5.2.1 Including parasites in species recovery plans

**Fig. 1:**
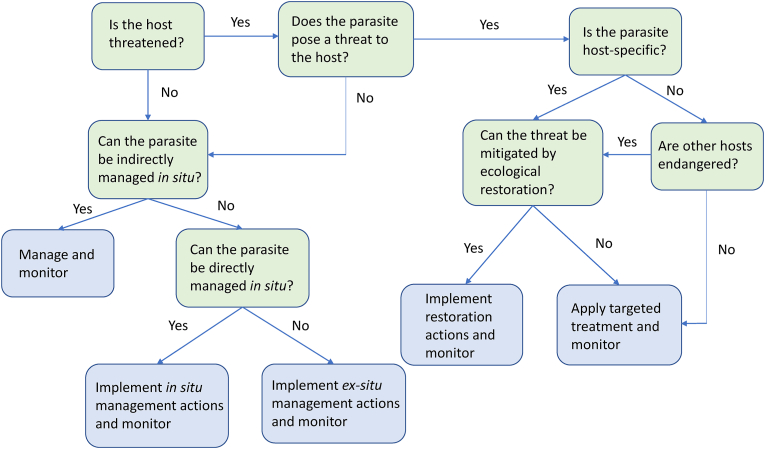
Decision tree for conservation management of threatened parasite species. See text for potential management actions. For other examples of decision trees, used in a more restricted fashion to assess parasite conservation status or to determine which parasites should be the focus of conservation actions, see Moir et al. (2011); Dougherty et al. (2016); Kwak (2018).

Species recovery plans aim to restore and maintain viable populations of threatened species (Doak et al., 2015). This typically involves management interventions either *in situ* (within the natural habitat of the species) or *ex situ* (relocation to areas outside the natural habitat), although the boundary between *in situ* and *ex situ* conservation is being increasingly blurred (IUCN/SSC, 2014). In cases where a parasite species but not its host is endangered, because of very low prevalence or a more restricted geographic range than the host, a parasite recovery plan needs first to consider whether the parasite can be managed indirectly by ensuring that host population density remains adequate for continued transmission, or whether more direct *in situ* or *ex situ* activities are required (Fig. 1). For example, Kwak et al. (2019) outlined procedures for the conservation of a flea, *Ceratophyllus* (*Emmareus*) *fionnus*, which is specific to the Manx shearwater (*Puffinus puffinus*). While the Manx shearwater has breeding colonies throughout the north Atlantic and is classified as Least Concern on the IUCN Red List, *C*. (*E.*) *fionnus* is apparently restricted to the Isle of Rùm, off the west coast of Scotland; Kwak et al. (2019) therefore proposed translocating the flea to other shearwater breeding colonies in the British Isles to create insurance populations.

In practice, conservation of parasite species is most likely to occur, not through stand-alone plans for the parasites themselves, but through their consideration as part of recovery plans for threatened host species. Many actions in current species recovery plans, particularly for *ex situ* conservation, increase the extinction risk for parasites, either directly, when hosts are treated to remove parasites which are considered detrimental to their health (Stringer and Linklater, 2014; Northover et al., 2017), or indirectly, if parasites are lost when hosts are maintained in cryobanks or zoos (Moir et al., 2012; Milotic et al., 2020) or translocated to new habitats (Northover et al., 2019).

A number of strategies can be used to reduce the risk of parasite extinction when implementing host recovery plans (Fig. 1) The first step should always be a risk assessment of the threat posed by a parasite to the viability of the host population; if host viability is not threatened then there is no requirement for parasite control and focus can return to whether the parasite can be managed indirectly through the host recovery plan or whether more direct actions are required. For parasites which may pose a threat to host populations and are also host-specific, then clearly the host must be conserved, because the parasite cannot survive if the host becomes extinct. However, rather than prioritizing host conservation over parasite conservation, a threatened host species and its host-specific parasites may be best considered as a threatened ecological community for the purposes of conservation (Moir et al., 2012; Barrera et al., 2022). For parasites which are host generalists, then it will be possible to use targeted parasite treatment for the threatened host, if alternate hosts are not endangered and are able to maintain the parasite life cycle. This could be achieved by using a narrow spectrum parasiticide and/or treating only hosts infected with the parasite, or by using a vaccine with limited cross-reactivity. When all host species are threatened, however, then they should also be regarded as a threatened ecological community along with the parasite species, and alternative methods of control considered.

When threatened parasites are regarded as a risk to host survival and some form of parasite control is required, Stringer and Linklater (2014) discuss a number of ways by which this risk might be minimized while reducing the possibility of parasite extinction, by attempting to restore the ecological conditions which maintain host and parasite in dynamic equilibrium. These include, for example, reducing exposure to environmental stressors that compromise the host immune response, using host translocations or improved connectivity among host populations to reduce inbreeding, re-introducing other (generalist) parasites to increase within-host competition, and reducing contact between wildlife hosts and potential reservoir hosts, such as livestock. As these ecological restoration strategies are less likely to have negative long-term consequences for the regulation of the host population and for ecosystem functioning, they should always be considered before treatment of the host with a parasiticide or vaccine.

Unfortunately, there is little indication that the conservation of threatened parasite species is currently given proper consideration in recovery plans for threatened host species. For example, the IUCN guidelines on the use of ex situ management for species conservation (IUCN/SSC, 2014) do not mention parasites or other dependent species. Similarly, the guidelines for re-introduction and translocation (IUCN/SSC, 2013) mention parasites only in the context of preventing disease transfer, except for the desirability of re-establishing the parasite fauna of hosts that have become extinct in the wild, and even then with the proviso that “… this should be subject to especially rigorous assessment of the risks … (as) … an apparently benign mutual relationship between host and parasite at source may change adversely for the host in the destination environment”. Pérez et al. (2013) state that the Iberian Lynx Captive Breeding Committee approved a proposal that the louse *Felicola isidoroi* be removed from wild lynx (*Lynx pardinus*) and transferred to captive lynx prior to insecticide treatment, but this seems to be an academic exercise as the louse has not been seen since 1997 (Pérez and Palma, 2001) and is presumed extinct (https://recentlyextinctspecies.com/phthiraptera-louse/felicola-isidoroi).

Although host recovery plans almost never consider the conservation value of parasites, there have been a few examples of unintentional parasite conservation during captive breeding and translocation programs for hosts. The tuatara tick, *Archaecroton sphenodonti*, has been translocated with its host (*Sphenodon punctatus*) onto an offshore island and a fenced reserve in mainland New Zealand (Moir et al., 2012); the beaver beetle (*Platypsyllus castoris*) was reintroduced to a number of countries across Europe with European beaver (*Castor fiber*) (Jørgenson, 2015); and three chewing louse species (*Ardeicola nippon, Colpocephalum nipponi*, and *Ibidoecus meinertzhageni*) were found on captive bred crested ibis, *Nipponia nippon* (Gustafsson et al., 2021). While these examples demonstrate that it is feasible to provide protection for both hosts and their parasites in species recovery plans, they also sound a note of caution. In the case of *A. sphenodonti*, tick abundances have declined dramatically in both destination sites, suggesting that tuatara densities in the founder populations were too low for successful transmission (Moir et al., 2012). Parasite conservation, therefore, is not simply a matter of stopping or adjusting routine antiparasitic treatment of hosts; effective conservation of parasites within a host recovery plan will require active consideration of the host and ecological factors that are required to maintain parasite transmission.

#### 5.2.2 Including parasites in ecosystem-centred conservation planning

Ecosystem-centred conservation refers to conservation actions directed above the species level or, for our purposes, above the level of the host-parasite community, and includes the assessment and management of protected areas and reserve networks. Ecosystem-centred approaches to conservation aim to sustain a representative sample of biodiversity, and the ecosystem processes they support, in a particular region (Keith, 2009; Wilson et al., 2009). These approaches are therefore assumed to act as surrogate restoration plans for species which are unknown or poorly understood (Noss, 1996) and for this reason have sometimes been seen as the most effective strategy for the conservation of parasite species (Gompper and Williams, 1998; Gómez and Nichols, 2013).

There is some evidence to support the effectiveness of ecosystem-centred conservation in protecting parasite species, with a number of studies reporting greater species richness, prevalence and/or abundance of aquatic parasites in marine protected areas than in unprotected sites (Loot et al., 2005; Lafferty et al., 2008; Wood et al., 2013). The extent to which these findings can be generalized, however, is not known. The most frequently used criteria when identifying areas for conservation of biodiversity are the presence of species of conservation concern (typically identified using the IUCN Red List) and sites in which individuals of threatened and/or migratory species congregate, such as nursery habitats and migration routes (Asaad et al., 2017). Neither of these criteria provides any information on the ecological conditions required for the successful transmission of threatened parasites (or, indeed on the likelihood that conservation efforts will work to protect the target species or other ecosystem assets; Wilson et al., 2009). A more useful criterion for identifying conservation areas, and one that is frequently applied to assess whether they are fulfilling their function, is ecological integrity, defined as the maintenance of species composition, diversity and functional organization (Parrish et al., 2003; Nicholson et al., 2009; Lee and Abdullah, 2019). While this should theoretically include an assessment of parasite diversity, given the importance of parasites in contributing to ecosystem function, in practice measures of ecological integrity are rarely quantitative and when they do assess species interactions, these never include parasitism (Nicholson et al., 2009; Lee and Abdullah, 2019).

## 6 Conclusions

Much more attention is now being given to the concept of parasite conservation, but this has yet to be translated into conservation actions. The implementation of on-ground parasite conservation is a priority if we are to move beyond a myopic focus on the conservation of charismatic vertebrate species to the preservation of healthy, functioning ecosystems that continue to provide the goods and services upon which human society depends. Perhaps the biggest challenge to be overcome is to improve the parasitological knowledge of conservation researchers and managers, and the general public, and to change the negative responses that parasites invariably provoke. We need to celebrate more widely the amazing diversity of parasites and the fundamental roles they play within ecosystems.

With our current understanding of the importance of parasites in maintaining the health of individual hosts, regulating host populations and mediating energy flow through trophic levels, what could have been done differently in the Californian condor conservation project? An initial step would have been to list the host-specific louse, *C. californici*, with the same threat status as its host, to ensure legal protection under the U.S. Endangered Species Act. The first question that should have been asked when conservation management was implemented for the condor was whether parasites represented a risk for the captive breeding and release program. Bird lice are all chewing lice which feed on feathers, skin and occasionally blood. They rarely cause much harm to their hosts and there is no evidence that *C. californici* posed any threat to the well-being or fecundity of condors. This suggests that there was no need for routine treatment of captive birds to remove lice; a better approach would have been to monitor host health and only apply treatment if there was clear evidence that lice were adversely affecting survival or reproductive ability. A thorough and detailed risk assessment of the health implications of this parasite on its host could potentially have saved a species from extinction.

Beyond merely withholding insecticide treatment, a number of positive steps could also have been taken to ensure the survival of the louse, along with its host. If parasite abundance had been recorded on all captured birds, these data would have provided a complete census of the existing louse population, providing a baseline to measure conservation success. Birds in captivity could have been monitored regularly for lice and, when necessary, prevalence could have been increased by housing infested and uninfested birds together or by manual transmission of parasites. Efforts should have been made to ensure that the louse was present in all captive breeding populations and in a proportion of birds (commensurate with the target prevalence) released into the wild.

## Funding

Research by AJL in this field has been supported by grants from the 10.13039/501100001037Australia and Pacific Science Foundation. NJS is in part supported by a Foundational Biodiversity Information Programme (FBIP) large grant from the 10.13039/501100001321National Research Foundation (NRF) of South Africa (Grant no. 138573). Opinions, findings and conclusions or recommendations expressed in this paper are that of the authors alone, and the funding bodies accept no liability in this regard.

## Declaration of competing interest

The authors declare no conflict of interest.
